# Role of Government Financial Support and Vulnerability Characteristics Associated with Food Insecurity during the COVID-19 Pandemic among Young Peruvians

**DOI:** 10.3390/nu13103546

**Published:** 2021-10-09

**Authors:** Katherine Curi-Quinto, Alan Sánchez, Nataly Lago-Berrocal, Mary E. Penny, Claudia Murray, Richard Nunes, Marta Favara, Anisha Wijeyesekera, Julie A. Lovegrove, Victor Soto-Cáceres, Karani Santhanakrishnan Vimaleswaran

**Affiliations:** 1 Instituto de Investigación Nutricional (IIN), Lima 15024, Peru; mpenny@iin.sld.pe; 2 Grupo de Análisis para el Desarrollo (GRADE), Lima 15063, Peru; asanchez@grade.org.pe (A.S.); nlago@grade.org.pe (N.L.-B.); 3 Department of Real Estate and Planning, University of Reading, Reading RG6 6UD, UK; c.b.murray@henley.reading.ac.uk (C.M.); r.j.nunes@henley.reading.ac.uk (R.N.); 4 Oxford Department of International Development, University of Oxford, Oxford OX1 3TB, UK; marta.favara@qeh.ox.ac.uk; 5 Hugh Sinclair Unit of Human Nutrition and Institute for Cardiovascular and Metabolic Research (ICMR), Department of Food and Nutritional Sciences, University of Reading, Reading RG6 6DZ, UK; a.wijeyesekera@reading.ac.uk (A.W.); j.a.lovegrove@reading.ac.uk (J.A.L.); 6 Faculty of Human Medicine, National University Pedro Ruiz Gallo, Lambayeque 14000, Peru; sotocaceresvictor@gmail.com; 7 Institute for Food, Nutrition and Health (IFNH), University of Reading, Reading RG6 6AH, UK

**Keywords:** food security, social programs, low- and middle-income countries, COVID-19, malnutrition

## Abstract

Peruvian households have experienced one of the most prevalent economic shocks due to COVID-19, significantly increasing their vulnerability to food insecurity (FI). To understand the vulnerability characteristics of these households among the Peruvian young population, including the role of the government’s response through emergency cash transfer, we analysed longitudinal data from the Young Lives study (*n* = 2026), a study that follows the livelihoods of two birth cohorts currently aged 18 to 27 years old. FI was assessed using the Food Insecurity Experience Scale. Household characteristics were collected before and during the COVID-19 outbreak in Peru to characterise participants’ vulnerability to FI. Multivariate logistic regression was used to evaluate the association between government support and participants’ vulnerability characteristics to FI. During the period under study (March to December 2020), 24% (95% CI: 22.1–25.9%) of the participants experienced FI. Families in the top wealth tercile were 49% less likely to experience FI. Larger families (>5 members) and those with increased household expenses and decreased income due to COVID-19 were more likely to experience FI (by 35%, 39% and 42%, respectively). There was no significant association between government support and FI (*p* = 0.768). We conclude that pre-pandemic socioeconomic status, family size, and the economic disruption during COVID-19 contribute to the risk of FI among the Peruvian young population, while government support insufficiently curtailed the risk to these households.

## 1. Introduction

Peru is one of the countries in the world most affected by the COVID-19 pandemic, with the second highest death rate in the Latin American region reached by 17 March 2021 (153 deaths per 100,000 inhabitants) [1]. Since the start of the pandemic (March 2020), a strict national lockdown was imposed which lasted 107 days. During this period, people were only allowed to go outside if they worked in essential sectors, and to buy food and medicine. This, along with subsequent regional lockdowns and other measures to promote social distancing (e.g., restrictions in capacity at shops and restaurants), as well as the direct effect of COVID-19 on morbidity and mortality, has caused significant social and economic disruption [2]. Within the Latin America Region, Peru experienced one of the largest contractions of its labour force in 2020—employment records for the economically active population saw a reduction of 13% [3]. In the same year, real GDP was reduced by 11.5% [4], and household poverty increased from 20.5 to 34% [5]. 

This economic disruption has threatened the ability of households to access adequate nutritious food, increasing their risk of food insecurity (FI) and malnutrition [6]. Compounding the problem, Peru’s healthcare system was under pressure due to the pandemic, which had resulted in reduced access to primary health care systems and placed non-immediate life-threatening illnesses (such as non-chronic diseases) to the back of the queue. There were also school closures and the interruption of vital food-security support programs such as school meals. Moreover, changes to lifestyles, for instance, the reduction in physical activity, increased sedentarism, and changes in dietary patterns also augmented the risk of FI, malnutrition, and their related long-term consequences to health and human development among the young [7,8]. The impact of these compounding factors on FI depends on the resources and capacities of the families to face them, being the most vulnerable those who have limited assets and financial savings to adjust to economic shocks [3,9]. According to UNICEF, poverty among young Peruvians increased 13% due to COVID-19 during the period 2019–2020, resulting in them becoming a vulnerable subpopulation in the country [9].

In this context, the Peruvian government issued an emergency cash transfer programme (known locally as ‘*Bonos*’) during the national lockdown and targeting 70% of households, offering a single payment of PEN 760 per household (equivalent to 82% of the monthly minimum wage) [10]. Direct cash transfers such as Bonos are considered a good support mechanism to aid poor families in times of economic shock [11]; however, previous reports suggest that Bonos might have arrived too late to help poor families, or failed to reach the most vulnerable populations, questioning their potential effect [12,13]. In addition to Bonos, it has been argued that having participated in other existing social programs before the pandemic could have beneficial effects, such as poverty reduction and improved access to education and health, therefore strengthening the initial capacity of the most vulnerable population to mitigate the effects of COVID-19 on FI [14]. To our knowledge, the role of this government support to mitigate FI during the pandemic has not yet been fully studied in Peru, and little is known about how the pandemic is affecting FI among the young. 

This is the knowledge gap addressed in this study, which uses longitudinal data from the Young Lives Study (YLS) in Peru. The YLS is a birth-cohort study started in 2002 that follows the livelihoods of two cohorts currently aged 18 to 27 years old [15]. We investigate the profile of the participants of the YLS affected by FI during the pandemic taking into consideration their pre-pandemic sociodemographic household characteristics and other shocks that made them more vulnerable to FI. We also evaluate the role of Bonos and existing social programs running before the pandemic in mitigating FI. Additionally, we identify the characteristics associated with the population who received Bonos to know whether they successfully reach the most vulnerable families.

## 2. Materials and Methods

### 2.1. Study Design and Participants

This secondary analysis based on data from the YLS which is a longitudinal survey, established in 2002 following two cohorts of children in Ethiopia, India (Andhra Pradesh and Telangana), Peru and Vietnam: a younger cohort born in 2001–2002, and an older cohort, born in 1994–1995 [15]. The sample selection of the YLS was based on a two-stage procedure. In the first stage, 20 districts from the universe of 1818 districts were randomly selected, excluding the wealthiest 5%. In the second stage, in each district, 100 families who had a child aged 1 year old for the younger or 8 years old for the older cohort were randomly selected for data collection. Although the sample is not nationally representative because of the exclusion of the wealthiest districts, the sample has been found to capture the diversity of the country in terms of geography, ethnicity, and socioeconomic status [15]. Each cohort was visited in 2002, 2006, 2009, 2013, and 2016. By 2016, attrition rates had reached 8.2% and 14.1% for the younger and older cohorts (*n* = 2468), respectively. In 2020, due to COVID-19, the YLS decided to administer a phone survey in the four countries (“Listening to Young Lives at work COVID-19 survey”), with the objective of assessing the short-term impact of the pandemic. During the phone survey, a first follow-up call was made to obtain up-to-date contact information of the YLS sample and 90% of those participants observed in 2016 (*n* = 2229) were contacted. Interviews were administered over mobile phones by 14 trained interviewers. Responses were recorded using SurveyBe, a computer-assisted personal interviewing software. Our study sample consisted of 2026 respondents to the YLS phone surveys. 

Each round of the YLS protocol was approved by the Ethics Committee of the Oxford Department of International Development, University of Oxford, in the UK and Nutritional Research Institute in Peru (Instituto de Investigación Nutricional in Spanish). The YLS phone survey protocol code is 054-2020/CIEI-IIN, approved on 19 May 2020, in Peru, and ODID CIA-20-034 approved on 15 May 2020, in the UK. Informed consent was obtained from all subjects involved in the study. All respondents of the YLS phone survey received PEN 50 for their participation, and a consultation guide with general information about COVID-19 and public services that were available in the country during the lockdowns.

### 2.2. Study Variables

Sociodemographic baseline characteristics: 

The sociodemographic variables were identified at household and individual levels. These were considered as baseline characteristics and were collected in the last visit of YLS before the pandemic (2016). At household level, we included: (i) mother’s education categorised into None (no formal education), Primary education, and Above primary (i.e., secondary and tertiary); (ii) ethnicity categorised by mother´s native language as Spanish or those with an Andean or native language; (iii) family size was categorised as those with ≥5 or <5 members; (iv) presence of children under 5 years (yes/no), and older adults over 65 years (yes/no); and v) household wealth index, as a proxy of socioeconomic status, categorised into bottom, middle and top terciles. The index is a composite measure of household wellbeing based on the average of a housing quality index (quality of floor, wall, roof, and number of rooms per capita), and access to services index (access to drinking water, electricity, sewage, and type of fuel used for cooking) and a consumer durables index (radio, television, bicycle, motorbike, automobile, landline phone, mobile phone, refrigerator, and fan) [16]. In this group, we also included location (urban/rural) and Peruvian region (coast, highland, jungle). These variables correspond to the place of residence of the participants reported in the last YLS phone survey (November–December 2020). 

At the individual level, we included: (i) the history of any chronic pathology, mental or physical disability reported by the YLS participants in the year 2016, and (ii) the history of malnutrition, which includes stunting and overweight. Having a history of stunting was defined as those YLS participants who were stunted at age 8, 12, and 15 years, while excess weight was defined as having this condition when the younger and the older cohort were 15 and 19 years old, respectively. This malnutrition indicator was assessed using anthropometric data of weight and height measured in the years 2009, 2013, and 2016 using standardised procedures that are described in detail in a previous publication [14]. Based on the WHO growth standards (2006) and the WHO AnthroPlus software, we estimated the z-scores of body mass index-for-age (BMI) and height-for-age (15), and defined stunted as those participants with a height-for-age z-score lower than <−2 standard deviation (SD), while overweight was defined in the younger cohort as those with a BMI z-score higher than 1 SD, and a BMI greater than 25 kg/m^2^ in the older cohort. BMI was calculated by dividing weight in kilograms by height in meters.

Self-reported changes due to COVID-19:

These variables were collected by a phone survey interview during the pandemic (August-October 2020) and included: (i) changes in household expenses and household income; (ii) presence of at least one member of the family infected by COVID-19; (iii) whether the family received assistance from friends or relatives during the pandemic, and (iv) the job sector of the employed YLS participants. The job sector was divided into 6 categories: (1) No work, (2) Agriculture/livestock/forestry, (3) Financial activities/accommodation, (4) Construction/mining, (5) Trade, and (6) Other services. We also considered if any of the family members, including the YLS participants, lost their job due to the pandemic. Being employed was defined as those that had been working in the last 7 days prior to the interviews. This variable was collected during June–July 2020.

Government Social Protection Programs:

We considered the government scheme of emergency cash transfers or Bonos as the main protection program during the pandemic. In addition, we included a set of regular social programs established prior to the pandemic. Bonos were delivered as an emergency economic relief during the lockdown consisting of a unique payment of PEN 760 soles per household (equivalent to 82% of a minimum monthly wage). Bonos were allocated according to the vulnerability of the recipients and launched at different at different stages according to the evolution of the pandemic within the national territory. They included (i) Bono for poor-urban households (“Bono *Yo me quedo en casa*” released on 16 March 2020); (ii) Bono for vulnerable households with independent workers (“Bono Independiente” released on 27 March 2020); (iii) Bono for poor households in rural areas (“Bono Rural” released on 19 April 2020); and, (iv) Bono for poor households with informal workers or monthly remuneration below S/3000 (“Bono Familiar Universal” released on 5 May 2020). In addition, for non-vulnerable families, there was a fifth Bono for workers affected by the suspension without payment and whose gross remuneration was a maximum of PEN 2400, released on 13 April 2020. This is shown in Supplementary Figure S1. For this variable, participants of the YLS were asked if anyone in their families received Bonos by June–July 2020. 

For the existing social programs, we included the conditional cash transfer program called Juntos aimed at the poor families in rural areas, as well as other food aid programs such as Glass of Milk, Community Kitchen, Food for Work, and any other complementary meal program delivered at primary health care centers. We consider beneficiaries of these programs if a member of the YLS participant’s family was receiving benefits before the pandemic (year 2016).

Finally, we included a binary indicator identifying YLS participants who were in lockdown for the longest period between March and December 2020. This is because though the national lockdown lasted for 107 days, some areas remained in lockdown for a longer period (up to 199 days). 

### 2.3. Food Insecurity

The food insecurity status of participants during COVID-19 was assessed using the Food Insecurity Experience Scale (FIES). This is a validated scale developed by the Food and Agriculture Organization (FAO) as a result of the project Voices of the Hungry (VoH) [17]. The FIES measures the severity of FI based on eight self-reported yes/no questions corresponding to the previous 12 months. These questions assess food-related behaviours, capturing the experience of FI at different degrees of severity, ranging from being worried about the ability to obtain food, to compromising the quality and variety of food, reducing quantities, skipping meals, and experiencing hunger (See questions on Supplementary Table S1). The FIES questionnaire was administered by 14 trained interviewers as part of the Young Lives phone survey, between November and December 2020. The FIES raw score ranged from 0 to 8 points. The status of FI was defined as those with or without this condition (yes/no). Participants with a raw score higher or equal to 4 points were categorised as food insecure representing those with moderate and severe food insecurity. Based on the FAO guidelines [18], before using the raw score, we performed a statistical validation of the FIES data to ensure its quality and to test its consistency with the theoretical assumptions of the scale, using the Rasch model (performed in the R v 4.0.3 software). The values of infit and outfit, residual correlation between FIES items, and the reliability of the Rasch model were in the acceptable ranges (0.7–1.3 per item, <0.4 and >0.7, respectively), indicating a good fit to the model and good overall data quality (Supplementary Table S2).

### 2.4. Statistical Analysis

First, the bivariate association between FI and each of the baseline household characteristics were investigated using the chi-squared test for two mean comparison of proportions and the analysis of variance (one-way ANOVA) for more than two comparisons. Second, a multivariate logistic regression model was used to identify the characteristics of young people who are more likely to be food insecure and assessed the role of the government on FI (Model 1).

**Model 1.**

$$\;P\left(FI_{i}=1\right)=G\left(\alpha_{0}+X_{i}\Gamma_{1}+\alpha_{1}Bonos_{i}+\alpha_{2}SociProg_{i}+\alpha_{3}Lenght_{i}+Z_{i}\Gamma_{2}+\mu_{i}\right)$$

$P\left(FI_{i}=1\right)$ is the probability that the household of participant is food insecure; $Bonos_{i}$ takes the value of 1 if the household received a cash transfer from the government for COVID-19 relief and 0 otherwise; $SociProg_{i}$ takes the value of 1 if the household is beneficiary or has access to at least one existing social program, 0 otherwise; $Length_{i}$ is a binary variable that takes the value of 1 if the lockdown lasted for 199 days and 0 otherwise; $X_{i}$ is a vector that contains the baseline household characteristics; $Z_{i}$ is a vector that incorporates the variable changes due to COVID-19; and, $\mu_{i}$ is measurement error. We report results for three specifications that successively incorporate the variables: the first specification includes vector $X_{i}$ $Bonos_{i}$ and $SociProg_{i}$. The second adds $Length_{i}$, and the third introduces the variable changes due to COVID-19. We proposed the three separated specifications to analyse how the duration of lockdown and changes during the pandemic could affect this relation. Additionally, we used an auxiliary multivariate logistic regression to identify the baseline household characteristics that affects the probability to receive Bonos in the population under study.

## 3. Results

**Figure 1:**
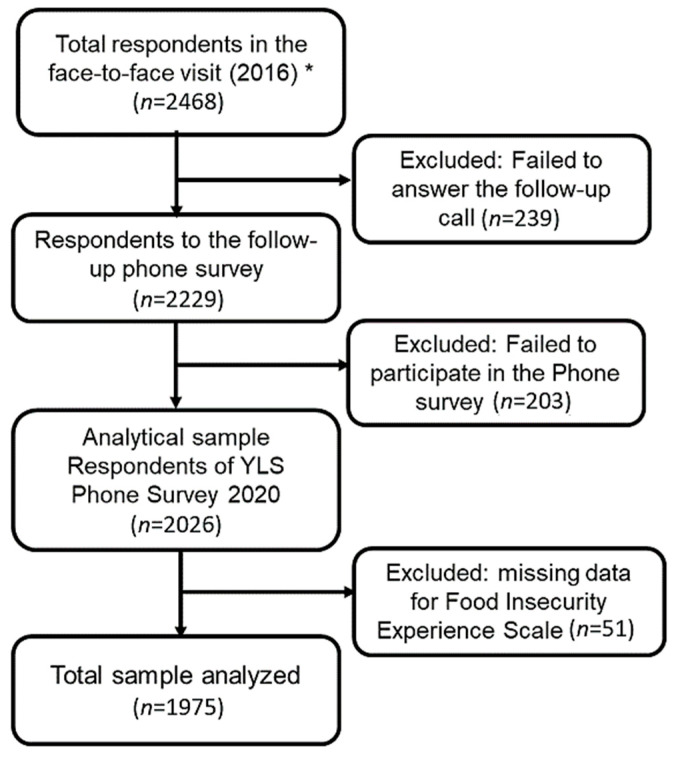
Flow chart of the analytical sample of participant of the Young Live Study in Peru (YLS). * Includes Peruvian participants from the younger (18–19 year.) and older cohort (24–27 year.) combined.

We analysed a total sample of 1975 participants from the YLS in Peru—younger and older cohorts combined, representing 97% of the study sample (*n* = 2026). We excluded 51 cases because of missing data in the main variable (FI). Our sample also represents the 80% of those participants observed in 2016. (Figure 1). 

The age range of the study population was 18 to 27 years with an average of 20.2 + 3.0. 77% of the sample corresponded to the younger cohort and the remaining 23% to the older cohort. The percentage of food insecurity (moderate and severe) was 24 % (95% CI 22.1–25.9%) with no statistically significant differences between the cohorts (22.4% vs. 24.5% in the older and younger cohort, respectively). 

### 3.1. Household Characteristics Associated with Food Insecurity Status

In Table 1 and Table 2, we present the results of the bivariate association of FI with baseline characteristics and changes due to COVID-19. Regarding the baseline characteristics, a higher proportion of FI was significantly associated (*p* > 0.05) with households with a lower level of maternal education (35% for those with no formal education), larger family size (>5) (29%), families with at least one child under 5 years old (27%), indigenous families (30%), those living in the rural (30%) and the jungle regions (29%), as well as those having low wealth index (32%). Participants who had a prolonged history of stunting at age 8, 12, and 15 years old were also associated with a higher proportion of FI (36%). Families that received any assistance from friends or relatives during the pandemic were associated with a higher proportion of FI (38%) as well as those families which included any member who became unemployed due to the pandemic (25%), as well as those working in primary activities (agriculture, livestock, and forestry).

### 3.2. Role of the Government Support and Vulnerability Characteristics Associated with Food Insecurity 

Table 3 presents the association between the government support and food insecurity. In model (1), the reception of Bonos was not associated with FI (*p* = 0.97). Likewise, having participated in any existing social program that had been running before the pandemic was not associated with FI (*p* = 0.18). These results remained consistent when the model was adjusted for lockdown length (Model 2) and subsequent changes to restrictions (Model 3). Additionally, household wealth and household size were consistently associated with food insecurity in all models (*p* < 0.05). According to Model 3, participants from households in the top tercile of wealth compared with those in the bottom tercile were around 49% less likely to be food insecure; while participants with families with more than 5 members, compared with those with less or equal than five, were 35% more likely to be food insecure; moreover, those with increased household expenses and decreased income due to COVID-19 were less likely to experience FI (39% and 42%, respectively). No association has been seen among other household vulnerability characteristics (place of residence, presence of child under 5 years, mother’s education and ethnicity) *p* < 0.05.

**Figure 2:**
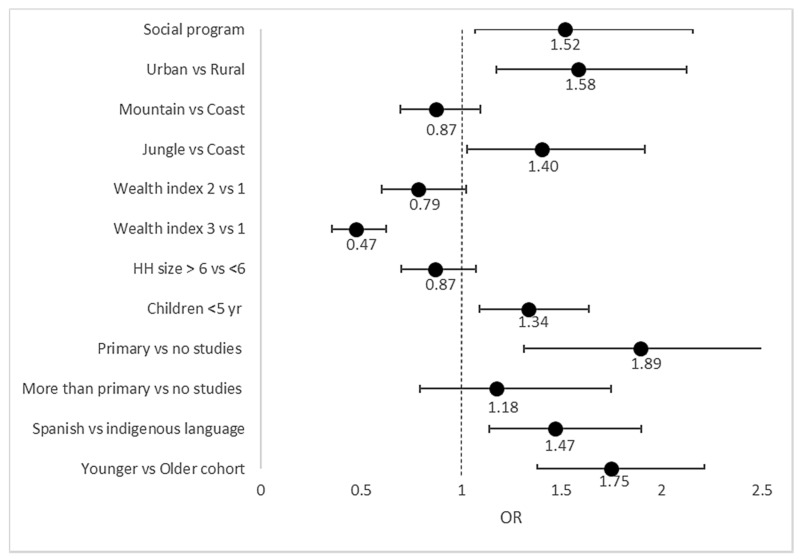
Household vulnerability characteristics associated with Bonos during COVID-19 from logistic multivariate regression model. Forest plot showing the household vulnerability characteristics associated with receiving any type of Bonos. The estimates are based on a multivariate logistic regression model. The values represent the estimated odd ratio (OR) for each characteristic, and the horizontal lines (whiskers) at both sides represents the 95% confidence interval of the OR. The vertical line represents the OR of 1 that indicates no association of the characteristic with the probability of receiving Bonos.

In the analysis of the characteristics of YLS participants who received Bonos (Figure 2), we found that families who were living in urban areas, and those who belonged to any of the social programs running before the pandemic, compared with those who did not, were 58% and 52% more likely to receive Bonos during the pandemic. In addition, participants living in the jungle region of Peru were 40% more likely to receive Bonos than those living in coastal regions, and families with any member under the age of 5 were 34% more likely to receive Bonos compared with those without children under 5 years old. Families considered non-indigenous compared with indigenous ones were 47% more likely to receive Bonos. In contrast, participants from households in the top tercile of wealth compared with those in the bottom were 53% less likely to receive Bonos. Finally, participants from the younger cohort (18–19 years) were 75% more likely to receive Bonos compared with the older cohort (24–27 years). 

## 4. Discussion

In this novel analysis involving the YLS cohorts, an average of 24% of the young population (18–27 years) experienced moderate and severe FI. According to our multivariate analysis, families in the top wealth tercile, larger families (> 5), and those that self-reported increased household expenditure and decreased income during COVID-19 were more likely to experience FI. Furthermore, the financial support from the government delivered during the first months of the pandemic (March to June/July 2020), was not associated with FI.

In relation to the prevalence of FI, we highlight that this was not homogenous among the study sample; sub-groups whose mother’s education level was low, indigenous and rural participants, and those with larger family size and low household wealth index prior to the pandemic had the highest prevalence of FI (29 to 36%). These sub-groups are more likely to have poor socioeconomic conditions, which is one of the main drivers of FI [19,20,21,22,23]. Additionally, they were consistently associated with disparities in the distribution of nutritional and health outcomes related to FI such as anaemia and stunting [21,24,25]. Regarding the results from the multivariate analysis, the positive relationship between family size (>5) and FI is expected, and consistent with previous studies. This is likely explained by an increased proportion of dependent family members (children, elderly) in larger households. [19,20,21,22]. This could be explained by the low capacity of these families to absorb any added economic pressures while catering for the needs of all members within their large family group in a context where COVID-19 has caused an important economic disruption to the already strained household’s income [5]. Our multivariate analysis also highlights the role of the pre-pandemic wealth index, which is negatively associated with FI, essentially capturing the relationship between material poverty and FI; as with family size, this relationship is to be expected in the absence of COVID-19, though it might be altered by it. Even after adjusting for these and household vulnerability factors included in the model, the observed relationship between FI and the perceived economic disruption of the household due to the pandemic—whether income decreased, or expenditure increased—confirms that COVID-19 had a direct impact on FI. In addition, although the area and region of residence, as well as the mother´s education level seem to be unconditionally associated with FI (according to Table 1 and Table 2), these individual associations become statistically insignificant once the wealth index is included in the model, which is expected since the wealth index summarises the poverty status of the household.

Our analysis also suggests that Bonos did not offset the FI experienced by families in the study sample. The objective of Bonos was to alleviate the economic impact of the national lockdown on family income in vulnerable populations. Previous reports suggested that problems to identify the most vulnerable populations as well as the delay in the delivery of Bonos could affect their effectiveness in the alleviation of poverty and FI. In line with this, to identify the vulnerable populations the government used the information of the socioeconomic classification from the National Household Targeting System (SISFOH by its acronyms in Spanish) [26]. However, the SISFOH was not updated and cases of filtering of government aid to non-vulnerable populations were reported [13]. Our analysis showed that families with high socioeconomic status were less likely to receive Bonos than those with the lowest socioeconomic status while urban populations and beneficiaries of any of the other social programs active prior to the pandemic were most likely to receive Bonos. This is in concordance with the criteria used by the government to identify the beneficiaries of Bonos [4]. Despite the government criteria, mothers of YLS participants with no education and indigenous families were less likely to receive Bonos during the pandemic. Ethnicity and low education have been associated with poor socioeconomic status, and we found a high proportion of FI (35% and 30%, respectively) in this sub-population. This finding is consistent with previous reports indicating problems in targeting the most vulnerable population using the SISFOH [13].

In relation to the delay in the delivery of Bonos, we found that by July 2020, 44% of the participants of the YLS´s families had received them. However, based on our data, we were unable to determine the number of participants who were entitled to receive Bonos. Nevertheless, according to the socioeconomic criteria, only 49% of the poor households (bottom wealth tercile) in our sample received Bonos by July 2020. This is in concordance with national data that reported delays in the delivery of Bonos. The main reason for this delay has been attributed to difficulties in reaching people using digital payments because only 38% of Peruvian adults have a bank account [27]. In addition, we can hypothesise that vulnerable households that received Bonos were not able to use the cash transfer to access food. This could be linked with the size of the transfer (82% of 1 minimum wage) which might have been insufficient to cover all the households’ needs. In absence of any other income sources, and the increase in household expenses during the pandemic, Bonos might have been used to cover other priorities such as health expenses instead of food expenses. Our findings show that families who reported reduction in income and an increase in expenses were 39% and 42%, respectively, more likely to experience FI. Moreover, most of the families on the bottom tercile of the wealth index reported a reduction in income (81%) and a 33.1% increase in their expenses due to the pandemic, and only half of them received Bonos by July 2020 (data not shown). Despite our limited data, we can speculate that for the most vulnerable population Bonos were insufficient or that they failed to receive them due to their invisibility in the government system, while less-vulnerable populations who received the Bono did not use it for food access. An analysis of how families use this kind of financial support would provide useful information about the family’s perceived needs and prioritisation scales, for instance whether they use Bonos to buy food or to satisfy other needs such as paying for shelter and health.

In addition to our main findings, we highlight that the FIES only measured one dimension of the FI, which is the financial security for food purchase. This is an important aspect, notwithstanding, a more comprehensive evaluation of FI including quality of diets, and the health/nutritional indicators [28,29] are needed to understand how COVID-19 is affecting the FI among the young population. This is particularly relevant for Peru, a country undergoing a rapid nutritional transition facing not only the increase in the prevalence of overweight conditions but also its coexistence with nutritional deficiency problems such as stunting and anaemia [30,31]. These factors could be worsened with the current pandemic. For instance, it is expected that there will be an increase in the prevalence of anaemia due to changes in diets [32]. According to the Peruvian bureau of statistics 14% of families from Metropolitan Lima and Callao were unable to buy sources of protein (meat, fish, and eggs) during the pandemic. Furthermore, changes in dietary patterns such as the lower consumption of minimally processed food, along with sedentary lifestyles, could contribute to an increase in the prevalence of excess weight [33].

In this study we were unable to collect the dietary and anthropometric data of the young population during COVID-19 because we changed from a face-to-face to a telephone survey due to social distancing measures. However, based on anthropometric data collected before the pandemic (2016) we found that YLS participants with a prolonged history of stunting during childhood and adolescence (8, 12, 15 years old), had a higher prevalence of FI, whereas being overweight during adolescence was not associated with FI. It is well known that stunting is the first manifestation of malnutrition during the first years of life, which reflects the cumulative effect of poor socioeconomic conditions that results in lack of access to adequate food and poor health conditions [34,35,36]. Therefore, in the context of the current pandemic, it is particularly important to prevent FI and stunting from infancy as well as in pregnant and childbearing women.

Finally, we can argue that the public policy related to FI must focus not only on providing financial security to purchase sufficient food, but also to complementary measures that ensure the access to good quality diets. This is particularly important for government-led social protection schemes such as food aid programs (e.g., Community Kitchen). These programs play a key role in mitigating FI in the context of the pandemic because they are supporting the most vulnerable in the population—a group that has increased due to the economic disruption caused by the pandemic [37]. Therefore, there is an urgent need to ensure that these programs provide good quality diets to promote a better nutritional and health status among the populations, otherwise these programs could unintentionally contribute to an increase in malnutrition. For instance, a previous study regarding the Community Kitchen scheme, reported a higher prevalence of obesity and metabolic disorders in its beneficiaries [38]. Furthermore, there is a need to reformulate these programs considering the aim to face the double burden of malnutrition in low- and middle-income countries [39,40,41]. In this context, improvements on program targeting and ensuring their use in accessing healthy food is needed to alleviate the impact of COVID-19 on FI of the most vulnerable population. 

### Limitation and Strengths

This is the first study that analyses the vulnerability characteristics of young populations and the role of government support on FI during the COVID-19 pandemic in Peru. We included longitudinal data to characterise the YLS population avoiding any simultaneity bias that mainly occurs when the exposure or risk factors and outcome are measured simultaneously. Additionally, standardised procedures and validated instruments, such as the FIES, were used for data collection reducing measurement bias. We also used a multivariate regression to analyse the independent association of each of the included variables with FI, reducing interpretation bias. 

A limitation of this study is a possible misclassification associated with attrition biases in the phone-survey, potentially resulting in an underestimation of the prevalence of FI. Nonetheless, our results were consistent with the prevalence reported in a previous study that analysed FI during the lockdown period in Peru [33]. We also recognise some limitations that may affect our results concerning the association between government support and FI. Firstly, we did not collect specific data regarding Bonos such as the number of participants that were selected to receive them, and the specific date when it was received. The lack of this data limits our capacity to explain our findings. Secondly, given our previous assumption that vulnerable YLS participants had received Bonos by the time of our analysis, the possible delay in their delivery might affect the validity of our results. Therefore, further studies are needed to establish a typical lag time between receipt of a cash transfer and its impact on FI before the role of Bonos and other support programs can be fully evaluated. Thirdly, the sample size is insufficient in detecting a 1.4% difference in the value reported in a previous study of the association between a cash transfer and FI [42]. Despite these limitations, our results suggest that Bonos did not reach the most vulnerable population in time, confirming its problems on focalisation [12].

## 5. Conclusions

During the initial phase of the COVID-19 pandemic in Peru (March–December 2020), 24% of the YLS population was affected by FI (moderate and severe). The characteristics associated with a higher vulnerability of FI were the lower household wealth, the larger family size, as well as the increase in household expenditure and income reduction during COVID-19. Furthermore, the financial support provided by the government during this initial phase did not alleviate or protect the families from FI. Delays in the delivery as well as the inadequate targeting of the most vulnerable population, could explain the inefficiency of this government support. In addition to our main findings, we highlight that despite the importance of the economic access to food as a driver of FI, it is important that current social protection programs such the cash transfer and food aid programs use complementary measures to promote food education and assure diet quality and active lifestyles to improve its effectiveness in alleviating FI in Peru which is facing the double burden of malnutrition.

## Figures and Tables

**Table 1:** Individual characteristics of the YLS participant by food insecurity status.

		Moderate and Severe FI (FIES ≥ 4)			
Total Sample		Overall (*n*)	No (%)	Yes (%)	*p*-Value χ2
		1975	76.00	24.00	
Sex of the YLS participant					
Male		1002	77.84	22.16	0.051
Female		973	74.10	25.90	
Type of cohort					0.348
Young Cohort (18–19 years)		1523	75.51	24.49	
Older Cohort (24–27 years)		452	77.65	22.35	
Mother Education					<0.001
None		199	64.82	35.18	
Primary education		718	74.23	25.77*	
Above Primary		1014	79.78	20.22 *†	
Ethnicity (Mother´s language)					<0.001
Any Andean/native language		559	70.13	29.87	
Spanish		1385	78.56	21.44	
Family size					
Less or equal to 5 members		1319	78.62	21.38	<0.001
More than 5 members		656	70.73	29.27	
Family composition					
Family with any children under 5 years.		739	72.94	27.06	0.014
Family without children under 5 years.		1236	77.83	22.17	
Family with any older adult (>65 years)		509	79.37	20.63	0.039
Family without older adult (>65 years)		1406	74.83	25.17	
Previous chronic pathology, mental or physical disability					
With pathology		118	73.73	26.27	0.551
Without pathology		1857	76.14	23.86	
History of malnutrition					
Stunting/short stature (8–12 and 15 years)		183	63.93	36.07	<0.001
Non-Stunting		1792	77.23	22.77	
Overweight		569	76.98	23.02	0.509
Non-Overweight		1392	75.57	24.43	

* Indicates significant difference with the first category; † indicates significant difference with the second category. The significance was assessed at *p* < 0.05 using a chi-squared test for two mean comparison of proportions and ANOVA with onferroni multiple comparison test for comparison of more than two categories.

**Table 2:** Household characteristics and changes due to COVID-19 by food insecurity status in the YLS participants.

	Moderate and Severe Food Insecurity (FIES ≥ 4)			
	Overall (*n*)	No (%)	Yes (%)	*p*-value χ2
Area of residence				
Urban	1625	77.35	22.65	0.002
Rural	350	69.71	30.29	
Region of residence				0.020
Coast	940	78.62	21.38	
Highland	748	74.47	25.53	
Jungle	287	71.43	28.57 *	
Wealth Index				<0.001
Bottom tercile	618	68.28	31.72	
Middle tercile	638	76.02	23.98 *	
Top tercile	702	82.91	17.09 †	
Self-reported changes due to COVID-19				
Increase in household expenses	1285	73.46	26.54	<0.001
Decrease in household income	1511	73.79	26.21	<0.001
Any member of the family with COVID-19	263	76.43	23.57	0.887
Unemployed due to COVID-19	634	75.00	25.00	0.001
Received assistance from friends/relative during COVID-19	370	62.16	37.84	<0.001
Job Sector of the YLS participants during COVID-19				
No work	373	83.11	16.89	0.001
Agriculture, livestock, and forestry	371	69.54	30.46 *	
Financial activities and accommodation	233	78.11	21.89	
Construction and mining	194	71.65	28.35 *	
Trade	340	75.88	24.12	
Other services	202	78.22	21.78	

* Indicates significant difference with the first category; † indicates significant difference with the second category. The significance was assessed at *p* < 0.05 using a chi-squared test for two mean comparison of proportion and ANOVA with Bonferroni multiple comparison test for comparison of more than two categories.

**Table 3:** Association of the Government support and household vulnerability characteristics with food insecurity among the YLS participants.

Variables	Model 1			Model 2			Model 3		
	OR	CI 95%	*p*-Value	OR	CI 95%	*p*-Value	OR	CI 95%	*p*-Value
The family received “Bonos” during COVID-19	1.00	0.80–1.26	0.971	1.00	0.80–1.25	1.00	0.97	0.77–1.27	0.768
Recipient of any existing social program before COVID-19	0.77	0.53–1.13	0.177	0.78	0.53–1.15	0.216	0.77	0.52–1.13	0.181
Longer period of lockdown (>199 days)	(...)	(...)		0.91	066–1.25	0.559	0.93	0.67–1.29	0.656
Household vulnerability characteristics									
Area of residence									
Urban	1.12	0.82–1.55	0.479	1.12	0.81–1.54	0.484	1.15	0.83–1.59	0.401
Region of residence									
Mountain	0.91	0.70–1.18	0.478	0.91	0.70–1.19	0.494	0.91	0.69–1.19	0.485
Jungle	1.13	0.79–1.59	0.506	1.16	0.81–1.67	0.423	1.15	0.80–1.65	0.463
Wealth Index									
Middle tercile	0.75	0.56–1.01	0.058	0.75	0.56–1.01	0.058	0.73 *	0.54–0.98	0.035
Top tercile	0.50 *	0.36–0.69	<0.001	0.50 *	0.36–0.70	<0.001	0.51 *	0.37–0.70	<0.001
Household size: more than five members	1.42 *	1.12–1.80	0.004	1.41 *	1.12–1.79	0.004	1.35 *	1.07–1.72	0.013
Presence of child under 5 years	1.16	0.92–1.46	0.215	1.16	0.92–1.46	0.216	1.15	0.91–1.45	0.244
Mother Education level									
Primary education	0.76	0.53–1.10	0.151	0.77	0.53–1.12	0.172	0.76	0.53–1.11	0.157
Above Primary	0.72	0.48–1.09	0.120	0.74	0.49–1.11	0.147	0.73	0.48–1.10	0.134
Ethnicity: Indigenous	1.23	0.93–1.64	0.150	1.25	0.94–1.67	0.128	1.20	0.89–1.61	0.226
Type of cohort: Younger cohort (18–19 years)	1.12	0.85–1.46	0.440	1.11	0.84–1.45	0.462	1.12	0.85–1.47	0.428
Self-reported changes due to COVID-19									
Increased household expenses	(...)	(...)		(...)	(...)		1.39 *	1.09–1.77	0.008
Decreased household income	(...)	(...)		(...)	(...)		1.42 *	1.06–1.90	0.018

* Indicates significant difference with the first category. Estimates based on a multivariate logistic regression model. Model 1 is adjusted for all the variables in the table, except “the length of lockdown” and “self-reported changes due to COVID-19”. In Model 2 we add to Model 1 the “lockdown length”; and in Model 3 we add to Model 2 the “self-reported changes due to COVID-19”. The reference group for area and region or residence are rural and the Coast, respectively; the bottom tercile for wealth index; < to 5 members for household size; “No” for the presence of children under 5 years; less than primary for mother´s education level; Spanish for ethnicity; older (24–27 years) for type of cohort. For changes due to COVID-19 the option “No” for increase in household expenses and decrease in household income. * Significant at 5% level.

## Data Availability

The individual data of the YLS participants in the four countries (Ethiopia, India, Peru, Vietnam) collected during the phone survey and previous in-person rounds, after de-identification, are publicly available via the UK Data Archive (study number 8678, DOI: 10.5255/UKDA-SN-8678-1; study number 8357, DOI: 10.5255/UKDA-SN-8357-1; study number 7931, DOI: 10.5255/UKDA-SN-7931-2; study number 6853, DOI: 10.5255/UKDA-SN-6853-3; study number 6852, DOI: 10.5255/UKDA-SN-6852-3; study number 5307, DOI: 10.5255/UKDA-SN-5307-3). The calculated food insecurity scale from Peru data is available upon request from the corresponding authors. The questionnaire is available at https://www.younglives.org.uk/.

## Supplementary Materials

The following are available online at https://www.mdpi.com/article/10.3390/nu13103546/s1, Table S1. Items of the food insecurity experience scale (FIES) applied in Peru. Table S2. Results of data quality assessment and validation using the Rasch Model. Figure S1. Timeline of the release of the government economic support (“Bonos”) during the study period of the COVID-19 pandemic (March to December 2020).
