# Supplementary material for: Role of Government Financial Support and Vulnerability Characteristics Associated with Food Insecurity during the COVID-19 Pandemic among Young Peruvians
Source: Nutrients. 2021 Oct 9;13(10):3546. doi: 10.3390/nu13103546 (PMC8539468; doi:10.3390/nu13103546)
Supplement: Supplementary file 1 [file nutrients-13-03546-s001.zip › nutrients-1382372-supplementary.pdf]

Supplementary Materials

Table S1. Items of the food insecurity experience scale (FIES) applied in Peru.

		The Food Insecurity Experience Scale (FIES 2020)<br>Young Lives Study Cohort
1	WORRIED	You or others in your household worried about not having enough food to eat because of a lack of money or other resources?
2	HEALTHY	In the past 12 months, was there a time when you or others in your household were unable to eat healthy and nutritious food because of a lack of money or other resources?
3	FEWFOOD	In the past 12 months, was there a time when you or others in your household ate only a few kinds of foods because of a lack of money or other resources?
4	SKIPPED	In the past 12 months, was there a time when you or others in your household had to skip a meal because there was not enough money or other resources to get food?
5	ATELESS	In the past 12 months, was there a time when you or others in your household ate less than you thought you should because of a lack of money or other resources?
6	RUNOUT	In the past 12 months, was there a time when your household ran out of food because of a lack of money or other resources?
7	HUNGRY	In the past 12 months, was there a time when you or others in your household were hungry but did not eat because there was not enough money or other resources for food?
8	WHLDAY	In the past 12 months, was there a time when you or others in your household went without eating for a whole day because of a lack of money or other resources?

.

Table S2. Results of data quality assessment and validation using the Rasch Model

country input data from R datafile

N complete non-extreme	1233
WN complete non-extreme	1233
N total	1978
N Any Missing	0
WN Any missing	0

								Validation			
Item	Severity	SE severity	Infit	SE infit	Outfit	N Yes on complete	Perc Yes on	INFIT < 0.7	INFIT 0.7 TO 1.3	INFIT HIGHER 1.3	OUTFIT <2
WORRIED	-3.02	0.08	1.04	0.04	0.78	992.00	80.45	NO	OK	NO	NO
HEALTHY	-1.20	0.08	1.02	0.04	1.41	593.00	48.09	NO	OK	NO	NO
FEWFOOD	-2.33	0.08	0.99	0.04	1.18	844.00	68.45	NO	OK	NO	NO
SKIPPED	0.94	0.10	0.95	0.07	1.04	209.00	16.95	NO	OK	NO	NO
ATELESS	-2.04	0.08	0.91	0.04	0.73	779.00	63.18	NO	OK	NO	NO
RUNOUT	1.60	0.12	0.82	0.08	0.79	141.00	11.44	NO	OK	NO	NO
HUNGRY	1.79	0.12	1.00	0.08	1.57	125.00	10.14	NO	OK	NO	NO
WHLDAY	4.27	0.27	1.11	0.23	7.06	15.00	1.22	NO	OK	NO	NO

Raw-score		Severity	Error	N cases	W cases	'cases relative
	0	-4.27	1.54	722.00	722.00	0.37
	1	-3.39	1.18	275.00	275.00	0.14
	2	-2.24	1.01	243.00	243.00	0.12
	3	-1.23	1.02	262.00	262.00	0.13
	4	-0.14	1.06	248.00	248.00	0.13
	5	0.98	1.05	112.00	112.00	0.06
	6	2.11	1.11	52.00	52.00	0.03
	7	3.61	1.38	41.00	41.00	0.02
	8	4.75	1.54	23.00	23.00	0.01

Reliab.	0.73
Reliab. flat	0.81

Distribution of valid responses

Num valid		Num cases	Pct cases	Wt cases	Wt pct	Pct if any v:	Wt pct if any valid
	0	0	0	0	0	0	0
	1	0	0	0	0	0	0
	2	0	0	0	0	0	0
	3	0	0	0	0	0	0
	4	0	0	0	0	0	0
	5	0	0	0	0	0	0
	6	0	0	0	0	0	0
	7	0	0	0	0	0	0
819,781,001,978,100,000,000							

Missing by item if any valid

Item	Num missir	Pct missing	Wt missing	Wt pct missing
WORRIED	0	0	0	0
HEALTHY	0	0	0	0
FEWFOOD	0	0	0	0
SKIPPED	0	0	0	0
ATELESS	0	0	0	0
RUNOUT	0	0	0	0
HUNGRY	0	0	0	0
WHLDAY	0	0	0	0

Residual correlation								Validation						
	HEALTHY	FEWFOOD	SKIPPED	ATELESS	RUNOUT	HUNGRY	WHLDAY	**HEALTHY**	**FEWFOOD**	**SKIPPED**	**ATELESS**	**RUNOUT**	**HUNGRY**	**WHLDAY**
WORRIED	0.075	-0.069	-0.037	0.022	0.040	-0.068	-0.065	OK	OK	OK	OK	OK	OK	OK
HEALTHY		0.064	-0.063	-0.043	-0.018	-0.112	-0.122		OK	OK	OK	OK	OK	OK
FEWFOOD			-0.023	0.107	-0.032	-0.070	-0.133			OK	OK	OK	OK	OK
SKIPPED				0.065	0.116	0.045	-0.062				OK	OK	OK	OK
ATELESS					0.047	0.017	-0.059					OK	OK	OK
RUNOUT						0.120	0.060						OK	OK
HUNGRY							0.002							OK

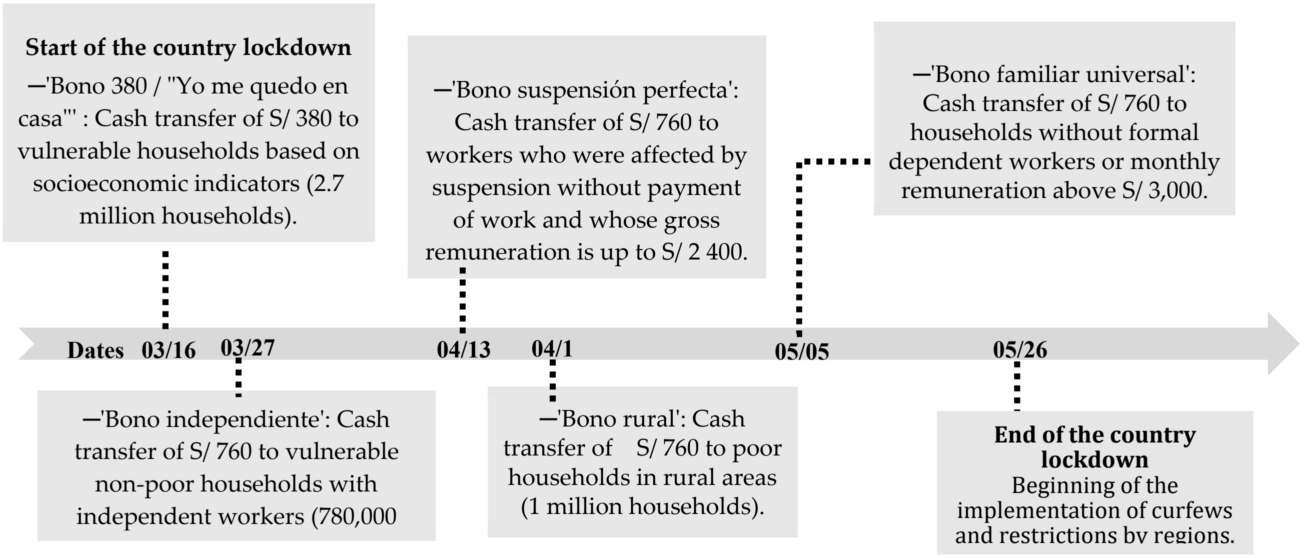

Figure. S1. Timeline of the release of the government economic support ("Bonos") during the study period of the COVID-19 pandemic (March to December 2020).
